# The TFE-induced transient native-like structure of the intrinsically disordered $\varvec\sigma_{ 4}^{ 70}$ domain of *Escherichia coli* RNA polymerase

**DOI:** 10.1007/s00249-014-0987-4

**Published:** 2014-09-27

**Authors:** Piotr Kaczka, Maria Winiewska, Igor Zhukov, Bożenna Rempoła, Krystyna Bolewska, Tomasz Łoziński, Andrzej Ejchart, Anna Poznańska, Kazimierz L. Wierzchowski, Jarosław Poznański

**Affiliations:** 1 Institute of Biochemistry and Biophysics, Polish Academy of Sciences, Pawińskiego 5a, 02-106 Warsaw, Poland; 2 Centre for Monitoring and Analyses of Population Health Status, National Institute of Public Health, National Institute of Hygiene, Warsaw, Poland

**Keywords:** Intrinsically disordered proteins, TFE-induced folding, NMR, ^15^N relaxation, Sigma-70, HLHTH motif

## Abstract

The transient folding of domain 4 of an *E. coli* RNA polymerase $\sigma^{70}$ subunit ($r{\text{EC}}\sigma_{4}^{70}$) induced by an increasing concentration of 2,2,2-trifluoroethanol (TFE) in an aqueous solution was monitored by means of CD and heteronuclear NMR spectroscopy. NMR data, collected at a 30 % TFE, allowed the estimation of the population of a locally folded $r{\text{EC}}\sigma_{4}^{70}$ structure (CSI descriptors) and of local backbone dynamics (^15^N relaxation). The spontaneous organization of the helical regions of the initially unfolded protein into a TFE-induced 3D structure was revealed from structural constraints deduced from ^15^N- to ^13^C-edited NOESY spectra. In accordance with all the applied criteria, three highly populated α-helical regions, separated by much more flexible fragments, form a transient HLHTH motif resembling those found in PDB structures resolved for homologous proteins. All the data taken together demonstrate that TFE induces a transient native-like structure in the intrinsically disordered protein.

**Electronic supplementary material:**

The online version of this article (doi:10.1007/s00249-014-0987-4) contains supplementary material, which is available to authorized users.

## Introduction

Intrinsically disordered proteins (IDPs) are involved in the essential processes of the living cell (Pancsa and Tompa 2012). Their sequences are generally enriched in polar and charged residues, but deficient in hydrophobic groups (Uversky 2002, 2011; Das and Pappu 2013). Free IDPs in a solution are highly flexible and do not display a stable secondary/tertiary structure (Dyson and Wright 2005; Tompa 2002). Under physiological conditions, IDPs sample a large conformational space, and some of the conformers, called a transient secondary structure, may resemble structured elements (Tompa 2005). In order to access the relationship between the structural dynamics of IDPs and the function, a continuum of thermodynamically accessible states has to be characterized in terms of both conformational free energy and interconversion time scales. Hence, a global and/or local flexibility provides the structural plasticity of IDPs, facilitating specific interactions with various partners (Wright and Dyson 2009), although the thermodynamics of partner-induced IDP folding must be linked directly to the intrinsic conformational properties (Uversky 2002, 2011; Das and Pappu 2013; Dima and Thirumalai 2004). IDPs, among others, are prominently represented in proteins associated with mRNA processing (Fu 1995; Buljan et al. 2012), apoptosis (Rautureau et al. 2010), signal transduction (Galea et al. 2008; Moncoq et al. 2003) and transcription regulation (Tompa 2002; Tompa 2005; Liu et al. 2006; Ozbudak et al. 2002; Wright and Dyson 1999).

In bacteria, two forms of RNA polymerase (RNAP), called holoenzyme (composed of α2ββ’ωσ subunits of a total weight of ~450 kDa) and a core (composed of α2ββ’ω subunits; ~400 kDa), are capable of transcribing DNA, but only the holoenzyme initiates transcription solely at the promoter sites. The identification of the σ-factor as a dissociable RNA polymerase subunit (Burgess et al. 1969) suggested that RNA polymerase may recruit other σ-factors to switch on particular regulons (Losick and Pero 1981). Seven different σ subunits have been identified in *Escherichia coli* (Maeda et al. 2000), each binding to the same core RNAP enzyme, thereby directing the transcription of a specific ensemble of genes. The actual model of bacterial transcription assumes a competition of sigma subunits for the limited amount of the core RNAP to which it can be bound (Maeda et al. 2000; Grigorova et al. 2006; Nyström 2004). In case of vegetative cell growth, σ^70^ predominates at a concentration exceeding that of the core RNAP (Grigorova et al. 2006; Jishage et al. 1996) and displaying the highest affinity of all sigma proteins to the core RNAP (Maeda et al. 2000), contributing to the transcription of 70 % of the operons (Raffaelle et al. 2005).

In a solution, the free σ^70^ subunit does not bind to promoter DNA because of direct intramolecular interactions between the N-terminal segment (region 1.1) and DNA-recognizing motifs located in the C-terminal part of the molecule (regions 3.2 and 4.2) (Camarero et al. 2002). The primary sigma factor of *E. coli* RNA polymerase (ECσ^70^) is a 613-residue protein of 70 kDa, belonging to the large family of sigma factors composed of four strongly conserved domains (Raffaelle et al. 2005). Its regions (2.4 and 4.2) specifically recognize the promoter sequences of expressed genes, located 10 and 35 bp upstream from the transcription initiation site, respectively. Some parts of the 4.2 region have also been identified as targets for transcription-activating or anti-sigma factors (Typas and Hengge 2006; Lambert et al. 2004; Patikoglou et al. 2007). Free σ^70^ in a solution must be regarded as carrying intrinsically disordered segments, as indicated by solution NMR studies on the segmental isotopic labeling of isolated region 4.2 of ${\text{EC}}\sigma_{4}^{70}$ (Camarero et al. 2002); this conclusion seems to be supported by the absence of a crystal structure of the free form of the sigma subunit, with only the exception of the solution structure of *Thermotoga maritime* $\sigma^{\text{A}}$ factor (TM $\sigma^{\text{A}}$) (Lambert et al. 2004).

At the moment, only two solution structures of ${\text{EC}}\sigma_{4}^{70}$ in complex with T4 Asia (record 1TLH) (Lambert et al. 2004) and *E. coli* regulator Rsd (2P7 V) (Patikoglou et al. 2007) are accessible in the Protein Data Bank. There is a known crystal structure of ${\text{EC}}\sigma_{4}^{70}$ in complex with a DNA-promoter fragment (3T72) (Blanco et al. 2011) and the structure of whole σ^70^ in the RNAP holoenzyme of *E. coli* (4IGC) (Murakami 2013), as well as its complexes with small-mass ligands (4KN7, 4KN4, 4KMU and 4JK2, 4JK1) (Molodtsov et al. 2013; Mechold et al. 2013). There are also the structures of homologous domains from thermophilic bacteria (Lambert et al. 2004; Campbell et al. 2002a, b). However, there are still no structural data concerning free ECσ^70^ in solution.

For our part, we have demonstrated that the population of secondary structure elements was substantially increased for $r{\text{EC}}\sigma_{4}^{70}$ in the presence of TFE (Poznanski et al. 2003). This generally agrees with the idea that TFE induces the closure of individual hydrogen bonds (Jaravine et al. 2001) and thus propagates the context-dependent formation of native-like secondary structures in polypeptides (Lawrence and Johnson 2002). Subsequently, we have monitored the TFE-induced conformational changes of $r{\text{EC}}\sigma_{4}^{70}$ with the aid of NMR spectroscopy (Kaczka et al. 2010). The drift of resonance signals in the ^1^H-^15^N HSQC spectra indicated that in a mixed water-TFE solution, a non-specific uniform build-up of helical structures takes place for a THE content smaller than 10 % (v/v). However, for a TFE content exceeding 10 %, non**-**monotonic changes were observed for numerous residues located in either the linker between domains 3 and 4 or the regions separating helical structures in the putative HLHTH motif (helix-loop-helix-turn-helix), namely G564-Y571 (L) and D581-T583 (T) (see PDB record 4igc). This supports the statement that at higher TFE concentrations, $r{\text{EC}}\sigma_{4}^{70}$ would tend to fold in a transient native-like 3D structure found in the crystal structure of the *E. coli* RNAP whole holoenzyme (Murakami 2013). Yet, does ${\text{EC}}\sigma_{4}^{70}$ in a solution resemble a sigma factor from *E. coli* RNAP much more than the homologous domains of *Thermus aquaticus* (Campbell et al. 2002) and *Thermus thermophilus* (Vassylyev et al. 2002), or the solution structure of *T. maritime* (Lambert et al. 2004), and that of a distant one, $\sigma_{ 4}^{ 5 4}$, from *Axuifex aeolicus* (2ahq) (Doucleff et al. 2005)? In this work, on the basis of further CD and NMR studies, we show a low-resolution model of *E. coli* $\sigma_{ 4}^{ 70}$ fold induced by a 30 % TFE, and we analyze the internal motions of the protein backbone with the use of ^15^N relaxation.

## Materials and methods

### Materials

The preparation of the recombinant ${\text{EC}}\sigma_{4}^{70}$ fragment, r${\text{EC}}\sigma_{4}^{70}$ (86 C-terminal residues of the *E. coli* σ^70^ preceded by a 21-aa segment carrying His_6_-Tag) was described previously (Poznanski et al. 2001). The protein was found to be chemically stable for a prolonged time in both aqueous acidic and mixed aqueous/TFE solutions.

### CD spectroscopy

Circular dichroism spectra were recorded on a Jasco J-815 spectropolarimeter equipped with a Peltier thermostatic cell holder. All the spectra were recorded for ~1.5 μM protein solution under a nitrogen atmosphere using a 10-mm path-length quartz cell. The exact protein concentration was determined for each sample based on UV absorption at 274 nm (*ε* = 1,400 M^−1 ^cm^−1^). Each CD spectrum was measured three times at 25 °C in the range of 195–270 nm. The contribution of the secondary structure elements was estimated with the aid of the CDNN program (Gerald 1997).

### NMR spectroscopy

The 2.5-mM protein samples were prepared in a 30 % (v/v) TFE/water binary system (10 % D2O) at pH 4.6. All $r{\text{EC}}\sigma_{4}^{70}$ NMR spectra were collected at 298 K on either a Varian Unity Plus 500 or Varian VNMRS 800 spectrometer, processed with the aid of NMRPipe (Delaglio et al. 1995), and analyzed using SPARKY (Goddard and Kneller 2002). Prior to the Fourier transformation, resolutions in indirect dimensions were increased by a π/3 shifted squared sine-bell weight function, followed by zero filling. The zero-order baseline correction was applied in all dimensions. ^13^C and amide ^1^H and ^15^N resonances were assigned using a combination of HNCBCA (Wittekind and Mueller 1993), CBCA(CO)NH (Grzesiek and Bax 1992) and C(CO)NH (Gardner et al. 1996) experiments. Additional HNHA (Kuboniwa et al. 1994) and HAHB(CO)NH (Wang et al. 1994) spectra enabled the assignment of the Hα and Hβ signals, while most of the remaining ^1^H aliphatic side-chain resonances were assigned using the H(C)(CO)NH spectrum (Grzesiek et al. 1993). Carbonyl ^13^C resonances were assigned using the combination of HNCO (Grzesiek and Bax 1992) and HN(CA)CO (Clubb et al. 1992) spectra. To overcome a problem with strong resonance overlapping, sequence-specific assignments were additionally confirmed by the inspection of sequential contacts identified in ^15^N-edited 3D-NOESY spectra. The assignments were deposited in BMRB (Entry 15975).

### Chemical shift analysis

Secondary chemical shifts, Δδ(*i*), were calculated for ^13^Cα, ^13^CO and ^1^Hα nuclei according to the reference values for random-coil chemical shifts corrected for sequence-dependent contributions (Schwarzinger et al. 2001). The population of helically folded regions in $r{\text{EC}}\sigma_{4}^{70}$ was then evaluated according to the concept of the chemical shift index (CSI) (Wishart and Sykes 1994). The helical population of individual residues, *p*_*α*_(*i*), was estimated as the ratio of Δδ(*i*)/Δδ_o_(*i*), where the reference values of secondary chemical shifts, Δδ_o_(*i*), were assumed to be 2.8, 2.3 and −0.4 ppm for ^13^Cα, ^13^CO and ^1^Hα, respectively (Wishart and Sykes 1994; Schwarzinger et al. 2000). In view of the consistency of the Δδ(*i*) distribution pattern for ^13^Cα, ^13^CO and ^1^Hα, the consensus local propensities toward a helical structure were expressed as the geometric average of the three descriptors referred to above (Kaczka et al. 2010). The population of helical structures were additionally estimated on the δ2D server (uk/d2D/ Jobs submitted: July 2014), the algorithm of which is based on the larger set of chemical shift data (^1^Hα, ^13^Cα, ^13^Cβ, ^13^CO, N and HN) (Camilloni et al. 2012). The protein backbone *φ* and *ψ* angles, and the square of the order parameter for the backbone amides, *S* ^2^, were additionally estimated with the aid of TALOS+ (Shen et al. 2009).

### ^15^N relaxation data

Relaxation parameters were measured at 298 K for a uniformly ^15^N-labeled protein sample dissolved in a mixed aqueous solution with 30 % TFE content (pH 4.6) on Varian UnityPlus 500, Varian VNMRS 600 and Varian VNMRS 800 spectrometers at 11.4, 14.1 and 18.7 T, respectively. The pulse sequences used for the determination of the ^15^N longitudinal (*R*_1_) and transverse (*R*_2_) relaxation rates were identical for all three spectrometers, being analogous to those originally reported (Farrow et al. 1994). For *R*_2_ measurements, a Carr-Parcell-Meiboom-Gill (CPMG) 180° pulse train with a refocusing delay of 650 µs was used during evolution (Meiboom and Gill 1958). Delays between proton π pulses, used for the suppression of cross-correlation effects between ^1^H and ^15^N nuclei (Kay et al. 1992), were 5 and 10 ms in *R*_1_ and *R*_2_ measurements, respectively. The recycle delay was kept as long as 2.5 s. One thousand twenty-four and 128 complex data points in time domains were collected in the hypercomplex mode. ^15^N decoupling during the acquisition was performed as a 3.2-kHz GARP pulse scheme (Shaka 1985). The relaxation rates were estimated using ten delays for *R*_1_ (0.01, 0.09, 0.17, 0.29, 0.41, 0.55, 0.69, 0.85, 1.01 and 1.25 s) and eight delays for *R*_2_ (0.01, 0.03, 0.05, 0.09, 0.13, 0.17, 0.21 and 0.25 s). {^1^H}-^15^N heteronuclear NOEs were measured according to the dynamic progressive saturation (Kowalewski 1978; Zhukov and Ejchart 1999) using the standard pulse sequence included in the BioPack (Varian Inc., Palo Alto, CA, USA) software. To overcome problems with peaks overlapping, the NOE intensities were assumed to be cross-peak’s heights. The *R*_1_ and *R*_2_ rates were then estimated by fitting an exponential decay curve in the form *I*(*t*) = *I*_0_exp(−*R*_*i*_ × *t*) implemented in SPARKY, where *I* is the signal intensity and *t* is the evolution time for appropriate magnetization. Severe signal overlapping limited the quantitative interpretation of relaxation parameters to only 84 out of 101 assigned backbone amide ^15^N nuclei.

### Analysis of the ^15^N relaxation data

The ^15^N relaxation data (*R*_1_, *R*_2_, ^1^H-^15^N NOE) were interpreted according to the reduced spectral density mapping, via sampling spectral density function, *J*(ω), at low (0), intermediate (*ω*_N_) and high (*ω*_H_) frequencies (Farrow et al. 1995).

$$R_{1} = \left( {\frac{{d^{2} }}{4}} \right)\;\left[ {J(\omega_{\text{H}} - \omega_{\text{N}} )\, + \;6J(\omega_{\text{H}} + \omega_{\text{N}} )} \right] + c^{2} J(\omega_{\text{N}} )$$

$$R_{2} = \left( {\frac{{d^{2} }}{8}} \right)\;\left[ {4J(0)\, + \;J(\omega_{\text{H}} - \;\omega_{\text{N}} ) + 3J(\omega_{\text{N}} ) + 6J(\omega_{\text{H}} ) + \;6J(\omega_{\text{H}} + \omega_{\text{N}} )} \right]\; + \;\left( {\frac{{c^{2} }}{6}} \right)[4J(0) + 3J(\omega_{\text{N}} )]\; + \;R_{\text{ex}}$$

$${\text{NOE}}\; = \;1\; + \;\left( {\frac{{d^{2} }}{{4R_{1} }}} \right)\;\left( {\frac{{\gamma_{\text{H}} }}{{\gamma_{\text{N}} }}} \right)\;\left[ {6J(\omega_{\text{H}} + \omega_{\text{N}} )\; - \;J(\omega_{\text{H}} \; - \;\omega_{\text{N}} )} \right]$$

1 $${\text{where }}c = \frac{{\omega_{\text{N}} \varDelta \sigma }}{\sqrt 3 },\;d = \left( {\frac{{\mu_{0} h}}{{8\pi^{2} }}} \right)\left( {\gamma_{\text{N}} \gamma_{\text{H}} } \right)\left\langle {\frac{1}{{r_{\text{NH}}^{3} }}} \right\rangle ,\quad\,\varDelta \sigma \; = \; - 160\,{\text{ppm}},\;r_{\text{NH}} = 1.02A,\;R_{\text{ex}} = \;a \times B_{0}^{2}$$

*J*(*ω*) is the spectral density of molecular motion at a given angular frequency, and the additional term *R*_ex_, which scales with the square of magnetic field, stands for the contribution of micro- to millisecond motions to *R*_2_ (Korzhnev et al. 2001; Stone et al. 1992). The above equation system can be resolved against *J*(*ω*) (Farrow et al. 1995), giving:

$$J(0.87\; \times \;\omega_{\text{H}} )\cong(0.8/d^{2} )\; \times \;\left( {\gamma_{\text{N}} /\gamma_{\text{H}} } \right)\; \times \;({\text{NOE}} - 1)\; \times \;R_{1}$$

$$J(\omega_{\text{N}} ) = [(4R_{1} - 7d^{2} )\; \times J(0.87 \times \omega_{\text{H}} )]/(3d^{2} + 4c^{2} )$$

2 $$J(0) = [(6 \times R_{2} - (9d^{2} /4 + 3c^{2} ) \times J(\omega_{\text{N}} ) - 39d^{2} /4 \times J(0.87 \times \omega_{\text{H}} )]/(3d^{2} + 4c^{2} )$$

The residues with possible contribution of conformational and/or chemical exchange to the transverse ^15^N relaxation were identified from the field dependence of *R*_2_–½*R*_1_ (Eq. 3), as those for which the estimated *R*_ex_ was identified, according to Peirce’s criterion (Peirce 1852) as outliers in the overall distribution of *R*_ex_.

3 $$R_{2} - \frac{{R_{1} }}{2} = \frac{{d^{2} }}{4}[2J(0) + 3J(\omega_{\text{H}} )] + \frac{{2c^{2} }}{3}J(0) + R_{\text{ex}} \cong \;\underbrace {{\frac{{d^{2} }}{2}J(0)}}_{{a_{0} }}\; + \;\underbrace {{\frac{{2c^{2} }}{3}J\left( 0 \right) + R_{\text{ex}} }}_{{a_{2} \times B_{0}^{2} }}$$

Since the studied protein was known to be highly unfolded (Poznanski et al. 2003; Kaczka et al. 2010), the standard Lipari-Szabo model-free approach (Lipari and Szabo 1982a, b), which attributes a global correlation time (*τ*_m_) to the overall rotational diffusion, was found inappropriate (Alexandrescu and Shortle 1994; Brutscher et al. 1997). Hence, an alternative approach of the direct fitting of the spectral density function (Eq. 4) to the *J*(*ω*) data sampled at 0, *ω*_N_ and 0.87×*ω*_H_ was used.

4 $$J(\omega ) = \frac{2}{5}\left[ {\frac{{S^{2} \times \tau_{\text{local}} }}{{1 + (\omega \times \tau_{\text{local}} )}} + \frac{{(1 - S^{2} ) \times \tau^{\prime}}}{{1 + (\omega \times \tau^{\prime})^{2} }}} \right]$$

where *S* ^2^ is the square of the residue-specific generalized order parameter for slow motions, 1/*τ*′ = 1/*τ*_local_ + 1/*τ*_e_, and *τ*_local_ and *τ*_e_ are the effective, residue-specific correlation times for slow (>1 ns) and fast (<100 ps) motions, respectively.

### Structural calculations

NOE-derived distance restraints were extracted from the ^15^N-edited 3DNOESY- HSQC (Talluri and Wagner 1996) (mixing time: 150 ms) and ^13^C-edited 3D-NOESY-HSQC (Muhandiram et al. 1993) (mixing times: 90 and 250 ms) experiments. The cross peaks in 3D-NOESY spectra were manually picked and assigned with the SPARKY program (Goddard and Kneller 2002), and their heights were then converted into distance restraints with the aid of the CALIBA procedure included in CYANA 2.1 (Guntert 2004). Additional constraints for backbone torsion angles were adopted from TALOS+ (Shen et al. 2009). Initial structural calculations were made with the simulated annealing (SA) protocol implemented in XPLOR 3 (Schwieters et al. 2003; Brunger 1992). The structure obtained with the homology modeling procedure was used as a template. The SA protocol started with 6-ns dynamics at 1,000 K, with dihedral angle restraints and NOE restraints scaled to 5 kcal mol^−1 ^rad^−2^ and 50 kcal mol^−1 ^A^−2^, respectively, and the van der Waals’ radii scaled down by a factor of 0.75. After that, the system was slowly cooled to 100 K during 3 ns, with the gradually increased terms for the repulsive van der Waals’ term (from 0.003 to 50 kcal mol^−1 ^A^4^) and for dihedral angles (200 kcal mol^−1 ^rad^−2^). Each SA cycle finished with 1 ns low-temperature evolution, followed by 1,000 steps of Powell energy minimization. This protocol was repeated 500 times, and a cluster of 11 lowest pseudo-energy structures was selected for further analyses. The final refinement was performed using additional constraints for experimental ^13^Cα, ^13^Cβ and ^1^Hα chemical shifts, using a chemical shift protocol (Kuszewski et al. 1995a, b). Finally, the structures were then inspected with PROCHECK-NMR (Laskowski et al. 1996), and all of them were subjected to further 5 ns molecular dynamic simulations in a water box using the Yasara2 force field implemented in the Yasara Structure package (Krieger et al. 2002). To prevent the momentary unfolding of the protein, and to mimic the helix-promoting propensities of a 30 % TFE solution, the medium-range (*i, i* + 3) constraints and angular restraints, imported directly from the SA protocol, were applied during the first 2 ns of simulations.

### Statistical tests

The relaxation parameters were analyzed in terms of their distributions, and the consistency of these data with normal distribution was initially tested with the aid of the Anderson-Darling goodness-of-fit test (Anderson and Darling 1952). Since some of the distributions significantly differ from the Gaussian one, the relaxation parameters obtained at different conditions (i.e., TFE concentration) or for different subsets of residues (e.g., His_6_-tag vs. HLHTH motif) were compared using the appropriate nonparametric Mann-Whitney *U* test (Mann and Whitney 1947). All tests were done with the aid of the Statistica package (2011).

### Computational methods

All the data analyses and presentations were performed using GnuPlot 4.6 (Williams and Kelley 2007).

## Results

### The HLHTH fold of $r{\text{EC}}\sigma_{4}^{70}$ in a solution

The protein was previously found soluble solely in a low-pH solution (Poznanski et al. 2003). Our previous studies demonstrated that the solubility of the protein substantially decreases above pH 4.5, which is far away from the theoretical p*I* of 9.1 ± 0.3, estimated as the average of nine different methods (Kozlowski 2007). This phenomenon should be directly addressed to the dissociation of Asp and Glu side-chain carboxyl groups at pH above 5. Dissociation of Asp and Glu side chains enables formation of numerous intra- and intermolecular salt bridges that compete with hydrophobic interactions (Poznanski et al. 2003). This destroys a hydrophobic core of the transiently folded protein, and the exposition of hydrophobic residues toward solvent results in intensive aggregation.

In order to stabilize the protein in a neutral solution, a number of solvent systems preventing the formation of salt bridges have been tested. Thus, 200 mM Na_2_SO_4_ and 1.8 M MgSO_4_ were found to minutely improve protein solubility at neutral pH, additionally stabilizing helical forms. However, limited protein solubility and the required salt concentration precluded the application of any NMR techniques. The high concentration of arginine (~1 M) also improved $r{\text{EC}}\sigma_{4}^{70}$ solubility (Buchner et al. 1992), but such a high concentration precluded even the CD monitoring of a putative Arg-induced secondary structure formation. Substantial progress was achieved with the use of zwitterionic non-detergent sulfobetaine stabilizers (NDSB). NDSB195 does not affect $r{\text{EC}}\sigma_{4}^{70}$ at low pH (Supplementary Figure S1a), albeit its 100 mM solution efficiently screens electrostatic interactions between dissociated side chains of Asp/Glu and those of Arg/Lys, and the protein solubility approached 50 μM at pH 7.7. Moreover, in the presence of 100 mM NDSB195, an increase in pH of $r{\text{EC}}\sigma_{4}^{70}$ solution results in a detectable increase of the estimated contribution of helical forms, which is clearly evidenced in CD spectra recorded at pH ranging from 2.6 to 7.5 (Supplementary Figure S2a), while the further increase of pH unfolds the protein. An optimal range of solution pH at which a protein is properly folded is a common protein property (Dill 1990; Copeland 2000). All CD spectra, analyzed together under the assumption of two-state equilibrium, show that, even at neutral pH, the protein is only partially folded. The maximal contribution of a putative folded state (approximately 50 %) was achieved at the physiological pH of 7.5 (see the insert in Supplementary Figure S2a). It should be stressed that 100 mM NDSB195 does not preserve the protein structure for $r{\text{EC}}\sigma_{4}^{70}$ concentrations exceeding 2 µM (see Supplementary Figure S2b for details), so this solvent system could not be used for heteronuclear NMR studies. Altogether, it clearly indicates that the previously proposed TFE-water binary solvent system is the most suitable for the NMR studies on $r{\text{EC}}\sigma_{4}^{70}$ structural preferences. The 30 % TFE concentration was found optimal, and the further addition of TFE did not result in visible changes of protein structure (Supplementary Figure S3). It should be mentioned that the CD spectrum of $r{\text{EC}}\sigma_{4}^{70}$ recorded in the presence of NDSB195 at pH 7.5 (Supplementary Figure S1a) resembles that recorded in TFE-water binary solvent (Supplementary Figure S3). This impression is strongly supported by the deconvolution of CD spectra against the contribution of secondary structure elements, populations of which were estimated with the aid of the CDNN program (see Supplementary Table S1).

### NMR resonance assignment and protein secondary structure preferences derived from CSI data in a 30 % (v/v) TFE solution

**Fig. 1:**
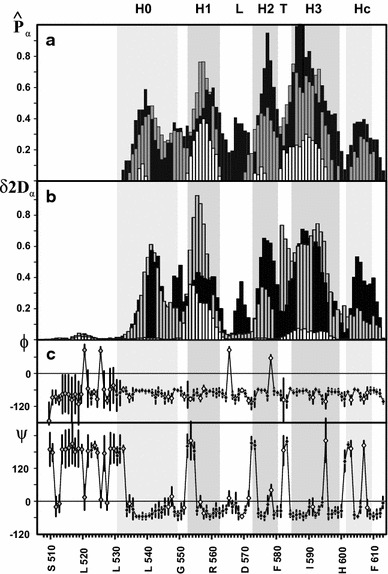
The distribution of a TFE-induced secondary structure along the $r{\text{EC}}\sigma^{70}_{4}$ sequence. The population of α-helical conformation was estimated from CSI data (**a**) and obtained from δ2D server (**b**) for an acidic aqueous solution (pH 2.8, *white bars*), 10 % (v/v) TFE (pH 4.5, *gray bars*) and 30 % (v/v) TFE (pH 4.6, in *black*). In parallel **c** the backbone angles phi (*φ*) and psi (*ψ*) were predicted with the aid of TALOS+ for $r{\text{EC}}\sigma^{70}_{4}$ in a 30 % TFE solution at pH 4.6. *Gray vertical strips* mark helical regions found in the structure of $\sigma^{ 70}_{ 4}$ homologs from thermophilic bacteria, and the *lighter strips* mark additional helical regions found in *E. coli* $\sigma^{ 70}_{ 4}$ in the RNAP complex. The *size of the error bars* represents the standard deviation from the average of the dihedral angles. *Gray* and *white markers* point out residues for which the conformation estimated by TALOS+ was reported as either dynamic or uncertain, respectively. Two of the latter, namely A594 and V606, were most likely wrongly predicted in non-helical conformation

The resonance assignments for $r{\text{EC}}\sigma_{4}^{70}$ at 30 % TFE (cf. Methods) were deposited in a BMRB database under accession no. 15975. A consistent pattern of the downfield shifts of ^13^Cα and ^13^CO resonances, accompanying the upfield shift of the ^1^Hα, was observed for numerous residues. The values of the score function, ${\tilde{p}_{\alpha } (i)}$ (see (Kaczka et al. 2010) for details) identify significantly populated helical patterns, located in the regions: 3.2 (L532-A542; H0), 4.1 (L551-F563; H1) and 4.2 (L573-L599; H2, H3). Another less populated fragment, S602-S609, is located in the C-terminal part of $r{\text{EC}}\sigma_{4}^{70}$ (Fig. 1a). The three clearly distinguished maxima coincide with the location of helical regions in the structures of homologous σ subunits (Lambert et al. 2004; Patikoglou et al. 2007; Blanco et al. 2011; Murakami 2013; Campbell et al. 2002), while the last one (S602–S609) could only be found in $\sigma_{4}^{\text{A}}$ of *T. maritime* (Lambert et al. 2004) and in *E. coli* $\sigma_{4}^{\text{A}}$ in complex with an Rsd regulator (Patikoglou et al. 2007) and in the recently solved structure of *E. coli* RNAP holoenzyme (Murakami 2013). The same trends, including both the location and population of helical structures, are also clearly visible when they are obtained with the aid of the δ2D server (Fig. 1b). The average population of helical structures in region 4.2 reaches 0.6 for a 30 % (v/v) TFE solution, while at 10 % (v/v) TFE it approached only 0.4 (Kaczka et al. 2010), respectively 0.33 and 0.37, when estimated using δ2d. The average population of helical structures for the whole protein was estimated to be 0.25 (Kaczka et al. 2010) and 0.38 for 10 and 30 % TFE solutions and respectively 0.25 and 0.26 when calculated using the δ2D approach. These values are close to the 0.35 estimated from the CD spectrum (see Supplementary table S1) and substantially higher than the 0.11 determined for the protein at low pH (Poznanski et al. 2003); 0.05 was determined using δ2D. This clearly indicates a considerable increase in the propensity of the $r{\text{EC}}\sigma_{4}^{70}$ backbone toward the α-helical fold in proportion to the increasing TFE concentration in an aqueous solution.

A comparison of the location of helical regions in the TFE-induced structure of $r{\text{EC}}\sigma_{4}^{70}$ with that found for its homologous domain of known structures (see gray vertical strips in Fig. 1, also highlighted in further figures) indicates that the C-terminal fragment of the H1 helix is considerably frayed, while H2 and H3, organized into the HTH motif, are in a similar location. The inspection of the estimated distribution of helical conformations along the protein sequence shows that the H3 helix is the most stable one, and it definitively does not propagate behind L598. The short helix H2 became significantly stabilized only at the highest TFE concentration (30 %), and the location of the loop region that separates H1 and H2 at a 30 % THE solution is similar to those seen in the crystal structures of the homologous proteins (Lambert et al. 2004; Patikoglou et al. 2007; Blanco et al. 2011; Murakami 2013; Campbell et al. 2002). It is worth noting that, according to the δ2D analysis (Fig. 1b), the residues located in the turn region of the HTH motif (D581-T583), which are in helical conformation at 10 % TFE, became non-helical at higher TFE concentration. This perfectly agrees with the break of monotonic drift in the HSQC spectra upon addition of TFE, previously observed for these residues (Kaczka et al. 2010). Altogether this proves that 30 % TFE does induce formation of a transient HLHTH structure, which is absent when the TFE concentration is lower than 15 % (Kaczka et al. 2010). The global analysis of chemical shift patterns performed with TALOS+ (Shen et al. 2009) also confirmed the presence of four helical regions in $r{\text{EC}}\sigma_{4}^{70}$ (Fig. 1c). The values of the φ and ψ backbone torsion angles were predicted as ‘good’ for 66 out of 103 analyzed residues. Among them, 32 are located in the putatively helical regions corresponding to the HLHTH motif. The conformation for the 20 residues located in the N-terminal region of the protein was consistently considered as dynamic. The next 17 residues, 9 of which are located within the HLHTH motif, were excluded from further analysis since their backbone conformation was predicted to be uncertain. Interestingly, the three N-terminal residues of the helix H1 (L551, T552, A553) were also predicted to be in non-helical conformation, which, according to the reported ‘good’ quality of the prediction, indicates that a TFE-induced helix H1 may be for $r{\text{EC}}\sigma_{4}^{70}$ shorter than these found in the known structures of homologous proteins. Similarly, residues Y571, T572 and G577, located at both of the termini of H2, are also predicted to be in non-helical conformation, which, together with the CSI data (Fig. 1a, b), proves that in ${\text{EC}}\sigma_{4}^{70}$, H2 is significantly shorter than those from homologous proteins (Lambert et al. 2004; Patikoglou et al. 2007; Blanco et al. 2011; Murakami 2013; Campbell et al. 2002).

### The internal motions of $r{\text{EC}}\sigma_{4}^{70}$ in a 30 % TFE solution derived from ^15^N relaxation data

**Fig. 2:**
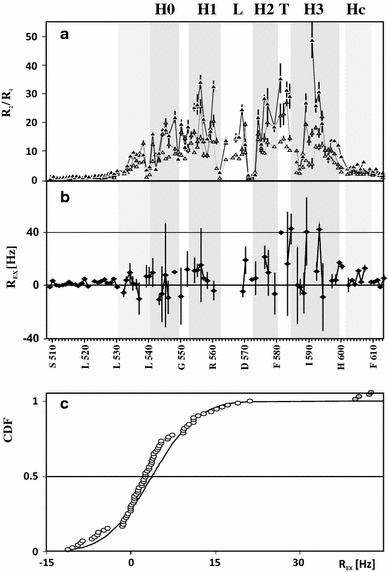
The distributions of the *R*_2_/*R*_1_ ratio along the protein sequence determined for $r{\text{EC}}\sigma^{70}_{4}$ in a 30 % TFE solution (**a**) and chemical exchange contribution,* R*_ex_, estimated at 11.4 T according to Eq. 3 (**b**). *White*, *gray* and *black markers* in *panel* **a** identify the values determined at 11.4, 14.1 and 18.7 T, respectively. *Vertical bars* represent estimates of the experimental error. *Gray strips* mark locations of the helical segments of the HLHTH motif. *Gray circles* mark outliers in cumulative distribution of *R*_ex_ (**c**)

The *R*_1_ and *R*_2_ relaxation rates, and {^1^H}-^15^N NOEs, were determined for $r{\text{EC}}\sigma_{4}^{70}$ in a 30 % TFE solution of 11.4, 14.1 and 18.7 T (see Supplementary Figure S4). As expected for substantially unfolded proteins, most of the {^1^H}-^15^N heteronuclear NOEs do not exceed 0.5, while the values expected for the limited internal mobility of N–H vectors in the folded proteins of a comparable size are of the order of 0.7 (Brokx et al. 2004). However, the residues located in the putative HLHTH region display visibly increased NOE values (Supplementary Figure S4c). Moreover, the variations in transverse relaxation rates along the protein sequence are consistent with the {^1^H}-^15^N NOE profile, clearly identifying two regions that differ significantly in their conformational flexibility. Elevated *R*_2_ values determined for residues R534-L598 (varying in the range of 15–45 s^−1^) strictly coincide with the location of regions displaying propensities toward a TFE-induced helical structure (Fig. 1). It is worth noting that residues S539, L540, T552, M561, R562, Y571, T572, L573, R586, I587 and R588, most of which are located in the putative HLHTH motif, display substantially decreased rates of backbone ^15^N transverse relaxation, which indicates their increased flexibility. The ratio *R*_2_/*R*_1_ is commonly used for identification of residues that experience motion of a timescale that differs from average molecular tumbling. Thus, decreased values of *R*_2_/*R*_1_, which reflect the variation in *R*_2_, together with negative values of heteronuclear {^1^H}-^15^N NOEs, thus again identify residues experiencing increased flexibility (see Fig. 2a). And high *R*_2_/*R*_1_ values point to residues with possible contributions of chemical exchange to the transverse relaxation. They may also be identified as those displaying substantial field dependence of *R*_2_–½*R*_1_. Both methods pointed to D581, R584, Q589 and K593 (Fig. 2a, b), which were also identified as outliers in the distribution of *R*_ex_ (Fig. 2c). Two of them are located in the turn region of the HLHTF motif, while the two other (Q589, K593) are located just in the middle of H3.

### Internal $r{\text{EC}}\sigma_{4}^{70}$ dynamics deduced from the reduced spectral density mapping

This analysis of ^15^N relaxation data does not require any assumption about the nature of molecular motion (Farrow et al. 1995). The distributions of *J*(*ω*) along the protein sequence are presented in Supplementary Figure S5A-C. The *J*(0), which is most sensitive for slow motions on the nanosecond time scale (Supplementary Figure S5c), follows the trend observed for *R*_2_ (Supplementary Figure S4c). The residues T537, E538, T544 T545, K557, D581-R584, E591, K593 and A594, for which *J*(0) substantially varies with the magnetic field, may undergo slow conformational exchange processes on the μs-ms time scale, the contribution of which to the rate of transverse relaxation (*R*_2_) scales with the square of magnetic field. The extremely low *J*(0) values observed for residues located in the N-terminal part of the protein of His_6_-tag and the linker fragment preceding Leu 528 (average of 0.24 ns/rad), together with visibly high correlation times for fast motions, *J*(*ω*_H_) (>20 ps/rad), show that this N-terminal part of the protein is almost unfolded. The same trend is observed for the C-terminal part of the protein (i.e., for residues succeeding HLHTH motif), albeit the visibly higher *J*(0) (average of 0.9 ns/rad) and slightly lower *J*(*ω*_H_) suggest that the motion of residues located in the C-terminal part of the protein is somehow restricted. The central part of the $r{\text{EC}}\sigma_{4}^{70}$ displays substantially higher values of *J*(0) and smaller values of *J*(*ω*_H_). This is indicative for the folded proteins; however, the values of *J*(*ω*_H_) higher than 7.5 ps/rad are indicative of residues experiencing significant internal flexibility (Viles et al. 2001). Interestingly, a few residues from the HLHTH motif are substantially more flexible than the others. All of them, R562, Y571, T572 and F573, are located in the loop region of the HLHTH motif. It should be thus concluded that this loop region undergoes fast conformational sampling. Finally, as expected, *J*(*ω*_N_) strongly varies with the strength of the magnetic field.

### Internal $r{\text{EC}}\sigma_{4}^{70}$ dynamics estimated according to the model-free approach

The simplest relaxation model describing the contribution of the slow and fast internal motions together with the order parameter for slow motion was used. In the case of poorly folded proteins, a correlation time for slow motions cannot be attributed to the correlation time characterizing the overall rotation of the protein, but must be regarded as a residue-dependent parameter. This makes the problem of fitting *J*(*ω*) to the relaxation data challenging. In general, only the simplest model (Eq. 4) was found reproducibly fitted upon several trial runs, while convergence of all others strongly depended on the starting values.

**Fig. 3:**
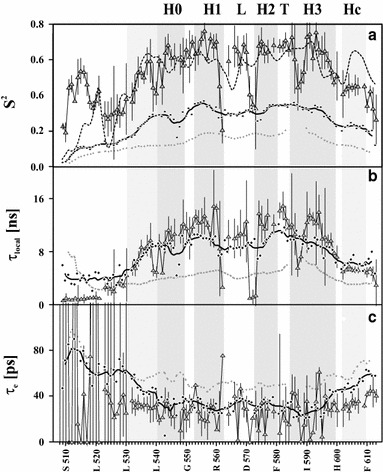
The interpretation of $r{\text{EC}}\sigma^{70}_{4}$ NMR relaxation according to the Model-Free approach optimized for IDP proteins (see Eq. 2a–c). *Gray vertical strips* mark the helical regions present in the HLHTH motif. Structured regions stabilized by TFE exhibit the increased values of the order parameter *S* ^2^, indicative of the build-up of an at least transient 3D structure. The distributions of both the generalized order parameter, *S* ^2^, and the corresponding slow-motion correlation times, τ_local_, indicate that the N-terminal 21-residue fragment is almost unfolded, and the mobility of the residues located in the central HLHTH motif are generally much more restricted than those located outside of this motif. The* thick broken line* follows the *S* ^2^ values estimated on the basis of the experimental chemical shifts of ^13^C, ^15^N, ^13^Cα, ^13^Cβ and ^1^Hα resonances according to the RCI algorithm included in TALOS+. For comparison, the data determined previously for low pH solution (*gray dotted line*) and 10 % TFE (*black line* following diamonds) are also presented

The distribution of the square of the generalized order parameter (*S* ^2^) along the protein sequence (Fig. 3a) demonstrates that the mobility of all residues located within the HLHTH motif is definitively much more restricted. It should be emphasized that the trend in *S* ^2^ variation, estimated from the chemical shift data (a thick broken line in Fig. 3a) follows that obtained directly from the ^15^N relaxation data. However, the local extrema do not strictly coincide with the expected location of helical regions indicated by CSI analysis (see Fig. 1a) and the predicted backbone angles (see Fig. 1b) identified in the structures of homologous proteins. The *S* ^2^ values for residues located in the HLHTH region (Table 1) are visibly smaller than those usually found for properly folded proteins (*S* ^2^ > 0.9), but exceed the value of 0.6 observed for the TFE-induced helix of calmodulin in 35 % TFE aqueous solution (Brokx et al. 2004).

**Table 1:** Relaxation parameters determined for $r{\text{EC}}\sigma^{70}_{4}$ in an acidic solution and for a 10 % and 30 % TFE solution at pH 4.55, calculated separately for the residues located in the N-terminal His_6_-tag and succeeding linker, in the HLHTH region, and in both regions flanking the HLHTH motif (others)

Conditions	*N*	*S* ^2^		*τ*_local_ (ns)		*τ*_e_ (ps)	
		Median	Mean	Median	Mean	Median	Mean
His_6_-tag + linker (residues preceding Leu 528)							
Low-pH	19	0.07 (0.05; 0.09)	0.07 (±0.03)	3.2 (2.3; 4.3)	4.1 (±2.8)	67 (61; 83)	72 (±15)
10 % TFE	21	0.11 (0.10; 0.12)	0.11 (±0.04)	4.0 (3.3; 4.6)	4.1 (±1.0)	66 (58; 72)	66 (±12)
30 % TFE	21	0.36 (0.30; 0.45)	0.37 (±0.10)	1.0 (0.8; 2.6)	1.7 (±1.1)	34 (16; 75)	97 (±143)
HLHTH (Ala 549-Arg 599)							
Low-pH	46	0.17 (0.16; 0.19)	0.18 (±0.03)	4.8 (4.3; 5.2)	4.9 (±0.8)	48 (45; 52)	49 (±5)
10 % TFE	49	0.32 (0.29; 0.34)	0.32 (±0.03)	9.5 (8.6; 9.9)	9.3 (±1.0)	31 (26; 36)	31 (±6)
30 % TFE	47	0.65 (0.58; 0.69)	0.61 (±0.12)	11.6 (9.9; 12.9)	10.6 (±3.4)	28 (16; 34)	26 (±16)
Others (L528-L548 and H600-D613)							
Low-pH	27	0.12 (0.11; 0.12)	0.11 (±0.01)	3.4 (3.0; 3.8)	3.5 (±0.5)	57 (55; 62)	59 (±7)
10 % TFE	30	0.23 (0.20; 0.28)	0.23 (±0.05)	6.8 (6.0; 8.5)	7.0 (±1.5)	46 (39; 53)	45 (±12)
30 % TFE	28	0.45 (0.43; 0.59)	0.49 (±0.10)	5.5 (5.1; 9.1)	6.8 (±2.3)	33 (28; 36)	32 (±8)

All the medians are accompanied by lower and upper quartiles, and corresponding mean values are accompanied by the standard deviations. Since some of the distributions deviated, according to the Anderson-Darling test, from the normal ones, they were compared using appropriate nonparametric tests (see Table 3 and “Materials and methods” for details)

The distribution of local correlation times for slow motions (*τ*_local_; cf. Fig. 3b) remains in complete agreement with the conclusion derived from the data presented in Figs. 1 and 2. Again, the four regions of $r{\text{EC}}\sigma_{4}^{70}$ (L532-D546, R554-M561, E574-D581 and R584-R599), characterized by the elevated values of *τ*_local_, are clearly visible. A considerable simultaneous decrease in *S* ^2^ and *τ*_local_ values observed for some residues located between D566 and L573 additionally supports the increased flexibility of this loop region. The median of 11.6 ns/rad (see Table 1) is visibly higher than that expected for the proteins of a similar size, which may result from increased viscosity of TFE-water mixed solvents (Olive et al. 1996). The possible contribution of slow conformational motions identified for residues T537, E538, T544 T545, K557, D581-R584, E591, K593 and A594 may also result in overestimation of the τ_local_ values.

The distribution of local correlation times for fast motions, τ_e_ (Fig. 3c), diverges strongly, and the values estimated for the HLHTH region are close to 30 ps.

### The low-resolution structure of $r{\text{EC}}\sigma_{4}^{70}$ in a 30 % TFE aqueous solution from the NOESY data

A total of 1,237 NOE contacts were assigned in ^13^C- and ^15^N-*edited* 3D-NOESY spectra recorded at a 30 % (v/v) TFE. This includes 778 intraresidual, 330 sequential, 106 medium-range (1 ≤ |*i* *–* *j*| ≤ 4) and 23 long-range (|*i* *–* *j*| > 4) ones. The number of medium-range contacts (see Table 2) significantly exceeds that previously identified at 10 % (v/v) TFE (106 vs. 15, respectively). However, most importantly, 23 long-range structural contacts between the regions that display a high population of secondary structure elements have been unequivocally assigned (see Supplementary Table S2 and Supplementary Figure S6). These contacts occur mainly between helical regions (e.g., H2–H3), but some involving loop regions (e.g., H1-L) can also be observed (see Table 2). Altogether, it clearly evidences the formation of at least a transient three-dimensional HLHTH structure. Such a small number of long-range contacts precluded any reasonable ensemble-based interpretation of NMR data (Rezaei-Ghaleh et al. 2012) and only representative low-resolution structures that generally agree with the CSI and NOE data.

**Table 2:** Long-range cross-peaks unequivocally assigned in ^15^N- and ^13^C-edited NOESY spectra recorded for $r{\text{EC}}\sigma^{70}_{4}$ at pH 4.55 in 30 % (v/v) TFE solution

	^15^N*-*edited 3D-NOESY mixing time: 150 ms	^13^C-edited 3D-NOESY mixing time: 90 ms	^13^C edited 3D-NOESY mixing time: 250 ms
H1-L		I565Hβ-R560Hβ2,3	
H1-H2			V576Hβ-M561Hα,γ3
H1-H3	E591HN-L559Hα	I587Hγ2-V558Hγ1	I590Hα,β-E555Hα
H2-H3	R584HN-G577HN; T583HN-E575Hα,β1,β2,γ1,γ2; T583HN-K578Hα,β1,γ,ε	I565Hβ-V576Hβ	I587Hα-V576Hγ1,γ2

As shown in Table 2 and Supplementary Table S2, the assignment of NOESY medium- and long-range cross-peaks, together with the relaxation data (Figs. 2, 3, S4, S5) and CSI analysis (Fig. 1), unequivocally indicate that $r{\text{EC}}\sigma_{4}^{70}$ contains four helical regions, separated by two loops and one short turn, similarly as has been found in the solution structure of homologous domains. The location of these helical regions was further constrained in the structural modeling to access the agreement between the TFE-induced transient fold of $r{\text{EC}}\sigma_{4}^{70}$ with NOESY-derived distance constraints (see Supplementary Table S3 for short statistics).

**Fig. 4:**
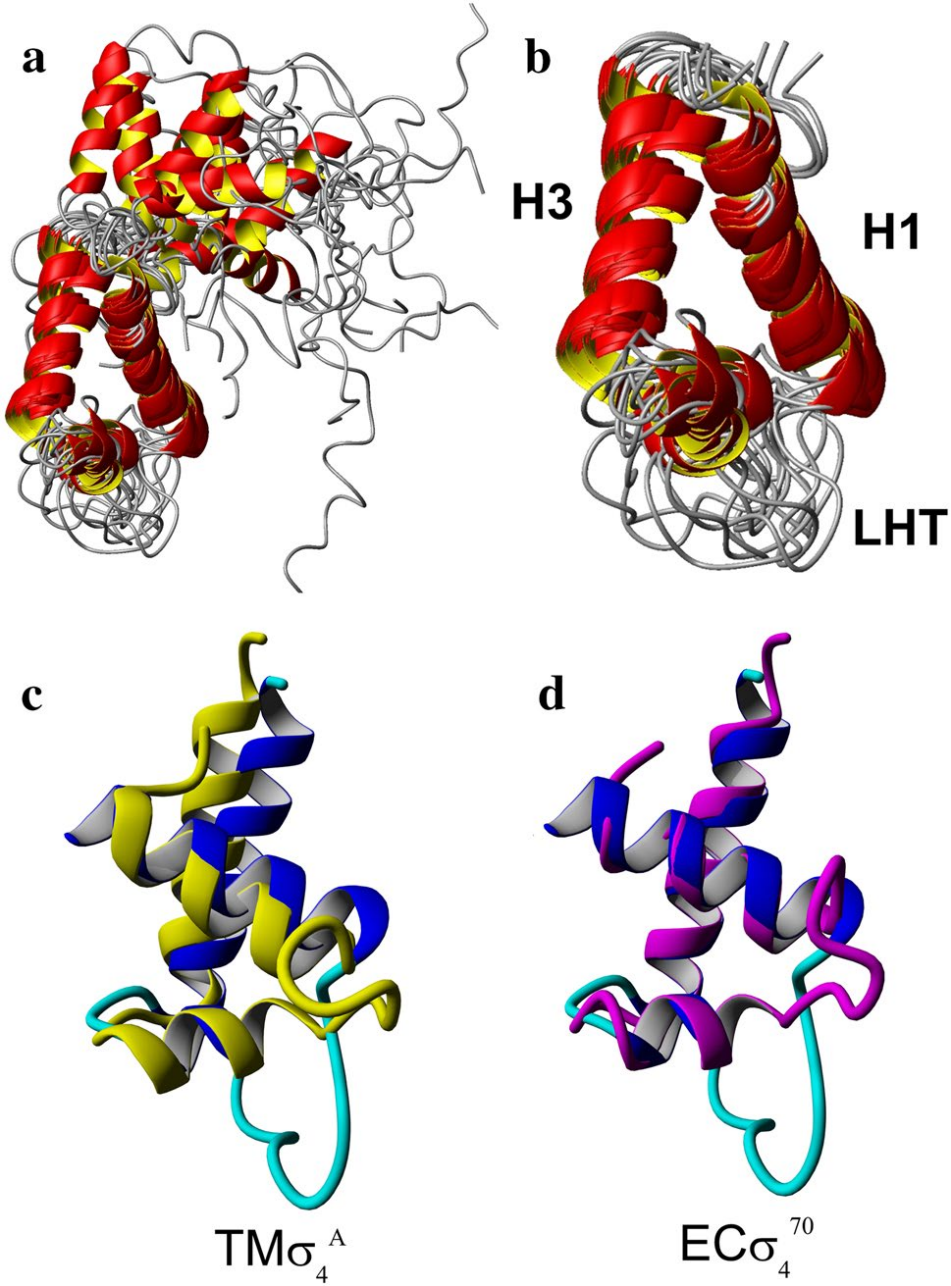
The transient solution structure of $r{\text{EC}}\sigma^{70}_{4}$ at a 30 % (v/v) TFE concentration. The ribbon representation of the ensemble of 11 lowest energy structures superposed for the H1 and H3 helical regions (**a**), HLHTH motifs of the latter (**b**) and superposition of the representative transient low-energy structure with the solution structure of *T. maritima* σ^A^ (**c**) and just published crystal structure of *E. coli* $\sigma^{ 70}_{ 4}$ (**d**) are presented

The ensemble of the resulting structures is presented in Fig. 4a. The arrangement of the H1 and H3 remains the most stable (see Fig. 4b), providing a kind of framework around which the more flexible LHT motif (i.e., a loop and short H2 region, followed by the turn) is organized in a form resembling those of TM $\sigma_{4}^{A}$ (Fig. 4c) and recently solved the whole RNAP holoenzyme for ECσ^70^ (Fig. 4d) (Murakami 2013). The average RMSD calculated for backbone heavy atoms for the H1 and H3 helical regions within the cluster of the 11 lowest (pseudo)energy structures was reduced to 2.1Å. The increased mobility of the LHT region may also be related to its physiological function (see the Discussion for details).

### The MD study of the structural stability of a TFE-induced HLHTH fold of r$EC\sigma_{4}^{70}$

The conformational screening was performed by 11 5-ns MD runs, and each of them started from one of the 11 lowest (pseudo)energy structures. Most of these trajectories preserved the initial protein fold, but during one of them a visible reorganization of the HLHTH motif, involving the simultaneous breaking of the H1 helix at R554-E555 and of the H3 helix at E591-A592, was observed. Both locations agree with the increased contribution of the chemical exchange process to ^15^N relaxation rates found for residues and E591-R593 (Fig. 2).

## Discussion

According to our earlier studies (Poznanski et al. 2003; Kaczka et al. 2010) and all the data presented in this work, $r{\text{EC}}\sigma_{4}^{70}$ in an aqueous solution must be regarded as intrinsically disordered, but capable of transient folding in the HLHTH motif in the presence of 30 % of TFE. In order to check to what extent the TFE-induced solution structure of $r{\text{EC}}\sigma_{4}^{70}$ resembles that of the homologous proteins, the most stable regions formed by the H1 and H3 helices of these two structures were compared (Fig. 4c, d). The backbone architecture of these two helices identified by us in the NMR-derived structure of $r{\text{EC}}\sigma_{4}^{70}$ is close to those of TM $\sigma_{4}^{A}$ in the solution and EC $\sigma_{ 4}^{ 70}$ in the crystal structure of the RNAP complex (Molodtsov et al. 2013), but the RMSD difference for the backbone atoms between the modeled solution structure and each of these two structures exceeds 10 Å. However, this difference is smaller than 2 Å, when calculated only for H1 and H3, confirming the high structural homology of these regions (see Fig. 4c, d). It has to be stressed that the sequences of all known reference $\sigma_{ 4}^{ 70}$ structures differ noticeably in their loop regions of the canonical HLHTH DNA-recognizing motif (I565-Y571 in *E. coli*). This loop is involved in the proper orientation of the recognition helix, interacting with the DNA major grove, and is also directly involved in an interaction with promoters (Campbell et al. 2002; Murakami et al. 2002).

### The internal dynamics of the TFE-induced fold of r${\text{EC}}\sigma_{4}^{70}$

The Anderson-Darling goodness-of-fit test demonstrated that the distributions of *S* ^2^, *τ*_local_ and *τ*_e_ estimated for $r{\text{EC}}\sigma_{4}^{70}$ are generally not Gaussian. The resulting medians accompanied by quartiles are presented in Table 1. For comparison, corresponding mean values and standard deviations are also presented. All the distributions were compared with the aid of the nonparametric Mann-Whitney *U* test.

**Fig. 5:**
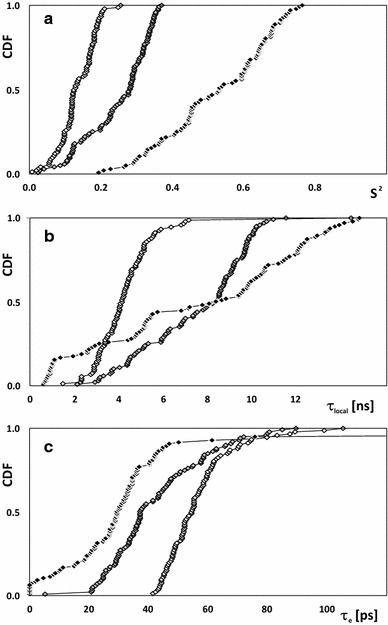
The cumulative distribution of generalized order parameter *S* ^2^ (**a**) and correlation times for slow (**b**) and fast (**c**) motions determined for ${\text{EC}}\sigma^{70}_{4}$ at low-pH solution (*open*), and 10 % (*gray*) and 30 % (*black*) TFE concentration

The global effect of the TFE concentration on ^15^N relaxation is clearly visible (Fig. 5). *S* ^2^ increases with the content of this agent (Table 1), reaching the maximum at 30 % TFE (v/v). Four helical regions indicated by the CSI analysis (see Fig. 1 for backbone angles estimated with TALOS+) agree with the distribution of relaxation parameters along a protein sequence (Fig. 3). It could thus be concluded that the extent of a transient fold of the protein increases upon TFE titration. According to the nonparametric Mann-Whitney *U* test (MW test), the distribution of *S* ^2^ differs significantly (*p* < 10^−6^) for all three protein regions, and the differences are statistically significant for all conditions tested (low pH, 10 % TFE, 30 % TFE, see Table 1 details and Table 3 for exact p-values for comparisons).

**Table 3:** The comparison of the estimated distributions of relaxation parameters determined for $r{\text{EC}}\sigma^{70}_{4}$ in various conditions made with the aid of the Mann-Whitney test

IDs	Compared distributions					
	HLHTH vs. others			TFE 30 vs. 10 %		
Conditions	Low-pH	10 % TFE	30 % TFE	His_6_-tag	HLHTH	Others
n1, n2	46, 27	49, 30	47, 28	21, 21	47, 49	28, 30
Parameter	*p*-values for comparison					
*S* ^2^	<10^−6^	<10^−6^	<10^−6^	1.6 × 10^−4^	<10^−6^	<10^−6^
*τ*_local_ (ns)	<10^−6^	<10^−6^	<10^−6^	0.13	<10^−6^	0.44
*τ*_e_ (ps)	<10^−6^	<10^−6^	1.7 × 10^−3^	0.32	1.6 × 10^−3^	<10^−6^

The IDs correspond to the data presented in Table 1

The medians for effective local correlation time for fast motions, *τ*_e_, calculated for the HLHTH region at 0, 10 and 30 % (v/v) TFE concentrations equal to 48, 31 and 28 ps, respectively, thus confirming a TFE-induced restriction of fast motions within the HLHTH motif of r${\text{EC}}\sigma_{4}^{70}$. The distribution of slow motions (*τ*_local_; Fig. 3b) remains in full agreement with the results obtained for *S* ^2^ and *τ*_e_. Again, the four regions of $r{\text{EC}}\sigma_{4}^{70}$ (L532-D546, A549-R560, V576- R582 and E585-R599) can be distinguished by the elevated *τ*_local_. The medians for *τ*_local_ are: 4.8, 9.5 and 11.6 ns for 0, 10 and 30 % (v/v) TFE concentrations, respectively.

The majority of these distributions differ significantly (see Table 3), proving that TFE addition does induce at least a transient protein structure, as indicated by elevated *S* ^2^ and restricted fast motions. A considerable simultaneous decrease in *S* ^2^ and *τ*_local_ values, observed for residues D566-L573, identifies a flexible structure located at the same protein sequence as the putative loop region of the HLHTH motif.

The above data clearly evidence that the addition of TFE to a protein solution stabilizes the HLHTH motif much more than that of the other regions of the protein, as proved by the direct comparison of the HLHTF motif with the flanking residues (others in Table 3). Generally, the distributions of *S* ^2^ and both correlation times estimated for residues located in the HLHTH motif significantly differ from those calculated separately for the peripheral residues located outside of this motif. However, it should be noted that all the TFE-related conformational changes generally lead to an increase in the population of the ordered structure, even for the residues outside of the HLHTH motif. This confirms that TFE acts as a non-specific structure-inducer, stabilizing the local secondary structures of folded proteins (Luo and Baldwin 1998; Bhakuni 1998; Shiraki et al. 1995), including α-helices (Nelson and Kallenbach 1989), β-turns (Cann et al. 1987) and β-hairpins (Ramirez-Alvarado et al. 1997). The thermodynamic effect of TFE should be connected with weakened non-local hydrophobic interactions, accompanied by slightly enhanced local ones, thus propagating context-dependent formation of a native-like secondary structure in polypeptides (Thomas and Dill 1993). This was reflected in an increase of the order parameter, *S* ^2^, accompanied by a moderate decrease of the local correlation time for fast motions, *τ*_e_. The structure-inducing effect observed for $r{\text{EC}}\sigma_{4}^{70}$ is definitively not uniform, but follows sequence-dependent propensities of the backbone already visible in a TFE-free low-pH protein solution (Poznanski et al. 2003).

All the results referred to above clearly demonstrate that IDPs, even in the absence of physiological partners, may obtain their functional folds. And these states can be sampled with a variety of commonly used stabilizing agents such as MgSO_4_, arginine, proline, sucrose, sarcosine, glycerol, NDSBs, TMAO or even by TFE, which also may act as a stabilizer (Luo and Baldwin 1998).

## Electronic supplementary material

Below is the link to the electronic supplementary material.

Supplementary material 1 (PDF 537 kb)
