# Supplementary material for: The TFE-induced transient native-like structure of the intrinsically disordered σ470 domain of Escherichia coli RNA polymerase
Source: Eur Biophys J. 2014 Sep 27;43(12):581–94. doi: 10.1007/s00249-014-0987-4 (PMC4236625; doi:10.1007/s00249-014-0987-4)

Table S1. Contents of secondary structure elements estimated for rECσ^{70}_4 in NDSB solution at neutral pH of 7.5, and in 30% TFE solution at moderately acidic pH of 4.5. The populations were estimated with the aid of CDNN program on the basis of three different spectral ranges. The consensus values and accompanying estimates of their standard errors are also presented.

Secondary structure	200-260 nm		205-260 nm		210-260 nm		consensus	
	TFE	NDSB	TFE	NDSB	TFE	NDSB	TFE	NDSB
Helix	35.3%	34.2%	37.1%	39.2%	35.5%	36.3%	36.0 (1.0)%	36.6 (2.5)%
Antiparallel	8.0%	8.6%	7.1%	6.7%	7.6%	7.5%	7.6 (0.5)%	7.6 (1.0)%
Parallel	8.1%	7.9%	8.0%	7.5%	8.2%	8.0%	8.1 (0.1)%	7.8 (0.3)%
Beta-Turn	16.5%	17.0%	16.1%	15.8%	16.4%	16.3%	16.3 (0.2)%	16.4 (0.6)%
Rndm. Coil	30.5%	28.9%	30.7%	29.2%	31.7%	31.2%	31.0 (0.6)%	29.8 (1.3)%
Total Sum	98.4%	96.5%	99.0%	98.5%	99.4%	99.3%	98.9 (0.5)%	98.1 (1.4)%

Table S2. Medium-range cross-peaks unequivocally assigned in ^{15}N-*edited* NOESY spectrum (mixing time: 150 ms) of rECσ^{70}_4 at pH 4.55, in a 30% (v/v) TFE solution

**N-terminus**	E529HN-M527Hα,Hβ2,Hβ3; L528HN -S525Hα; P531HN -M527Hα; A535HN -L532Hα; E538HN-S534HN,Hβ2,Hβ3; E538HN-D533Hα,Hβ3; A542HN-T537Hα,
**H1**	A553HN-L548HN,Hα,Hβ2,Hβ3,Hγ,Hδ1,Hδ2; A553HN-A549Hα; A556HN-A553Hα,Hβ; K557HN-A553Hβ; K557HN-R554HN; M561HN-K557Hγ2,Hγ3,Hδ2,Hδ3; M561HN-V558HN; G564HN-R560HN
**L**	E574HN-T572HN
**H2**	V576HN-L573Hβ3,Hγ,Hδ1,Hδ2; K578HN-E575Hα,Hβ2,Hβ3; Q579HN-E575Hβ2,Hβ3,Hγ3; Q579HN-V576HN; V582HN-K578Hα,Hβ2,Hβ3,Hγ2,Hδ2; V582HN-Q579HN
**T**	R584HN-F580Hβ2,Hβ3; R584HN-D581Hα
**H3**	I587HN-T583HN; K597HN-A594Hα,Hβ; H600HN-R596Hβ2,Hγ; S602HN-R599Hα,Hβ2,Hγ; S604HN-P601Hα,Hβ2,Hγ
**C-terminus**	L611HN-R608Hα,Hβ1,Hβ2,Hδ; D613HN-S609Hβ1,Hβ2

Table S3. Statistics of NMR restraints and the resulting structure.

NOE-derived constraints	
Unambiguous	854
Intraresidual	473
Intrerresidual	381
sequential (|i-j|=1)	287
medium-range (1< |i-j| ≤4)	71
long-range (|i-j| > 4)	23
Ambiguous	383
Intraresidual	305
Interresidual	78
sequential (|i-j|=1)	43
medium-range (1< |i-j| ≤4)	33
long-range (|i-j| > 4)	2
Predefined hydrogen bonds	60
Talos+ (φ, ψ)	75
Total number of experimental restraints	989
Mean pairwise RMSD for HLHTH region (A549-R599)	
backbone atoms:	3.67 (±0.82)
all heavy atoms:	4.86 (±0.91)
Mean pairwise RMSD for the whole protein (G508-D613)	
backbone atoms:	10.74 (±2.00)
all heavy atoms:	11.33 (±1.83)
Ensemble Ramachandran plot for the whole protein (G508-D613)	
most favored	61.5%
additional allowed	20.9%
generously allowed	14.4%
Disallowed	3.2%

Figure S1. CD spectra of rEC$\sigma^{70}{}_4$ in the presence of 100 mM NDSB195 recorded at neutral and denaturating conditions (A) and urea denaturation of NDSB195-induced fold (B). Reference CD spectra of low-pH (unfolded) and TFE-induced (folded-like) forms of rEC$\sigma^{70}{}_4$ are also presented.

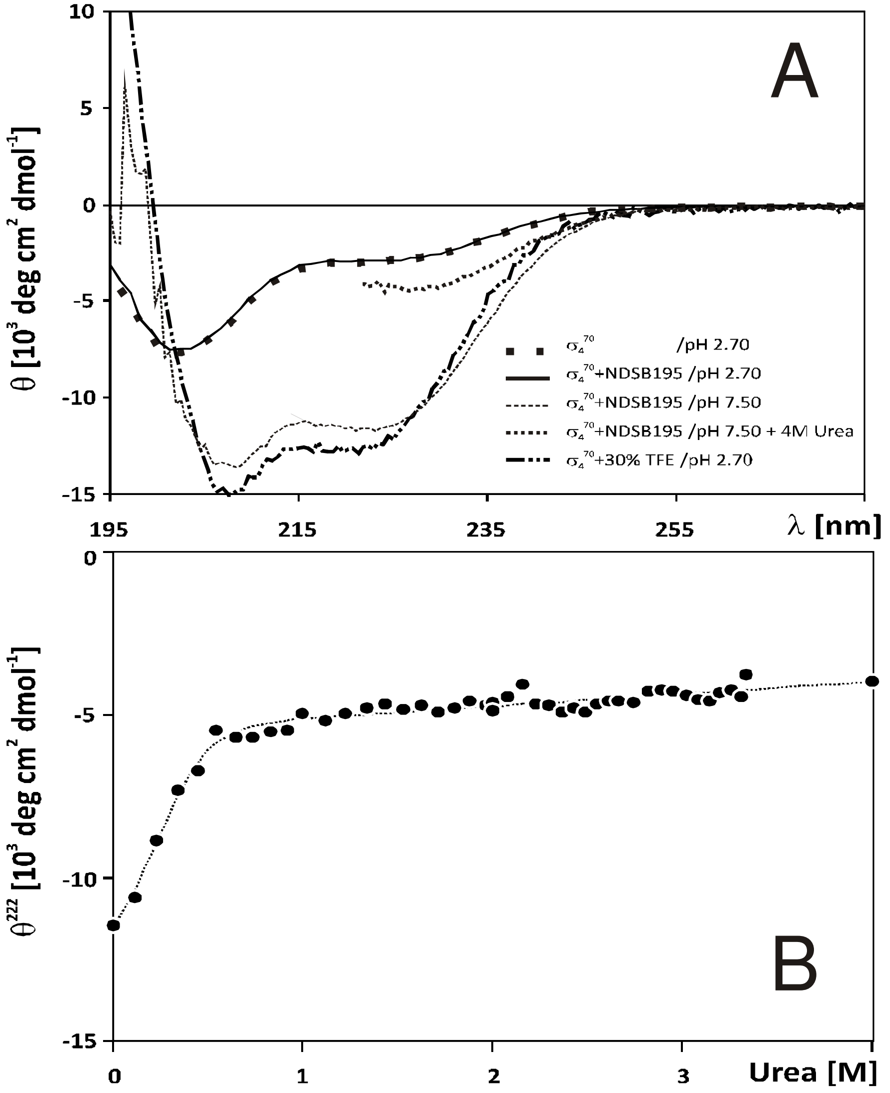

Figure S2. CD spectra of rECσ^{70}_4 recorded at increasing pH (A) and at increasing protein concentration in the presence of 100 mM NDSB195 at neutral pH (B). In the inset estimated pH-dependence of folded/unfolded states is presented.

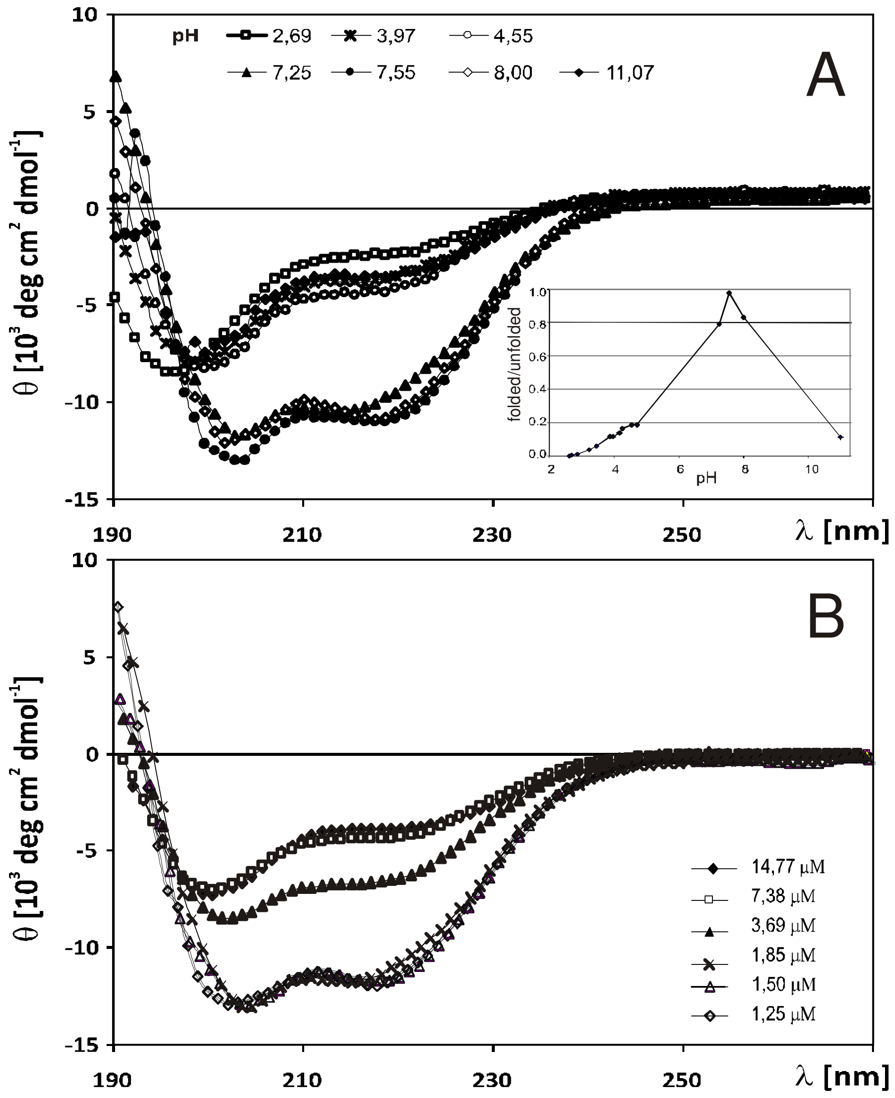

Figure S3. CD spectra of rECσ^{70}_4 recorded at increasing TFE concentration. In the inset population of folded form estimated according to the two-states model..

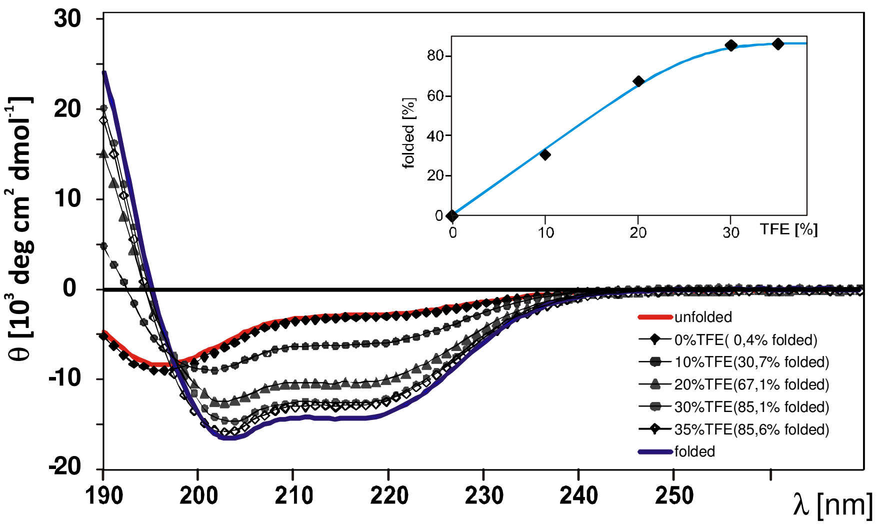

Figure S4. Longitudinal (R_1, A) and transverse (R_2, B) relaxation rates, and heteronuclear $\{^1H\}$-^{15}N NOEs (C) determined for the amide backbone nitrogen atoms. Values, represented as a function of rECσ^{70}_4 sequence, were estimated for a 30% (v/v) TFE solution of rECσ^{70}_4 in experiments carried out at 11.7 T (white triangles), 14.1 T (grey triangles) and 18.7 T (black triangles) (25°C, pH 4.6). Grey vertical strips mark helical regions found in the structure of σ^{70}_4 homologues from thermophilic bacteria, and the lighter strips mark additional helical regions found in E. coli σ^{70}_4 in the RNAP complex. Experimental errors are denoted by vertical lines. For comparison the analogous data (D-E) obtained previously for low-pH solution at 11.7 T (black triangles), and 10% TFE at 9.4 T (white squares) and 11.7 T (black diamonds).

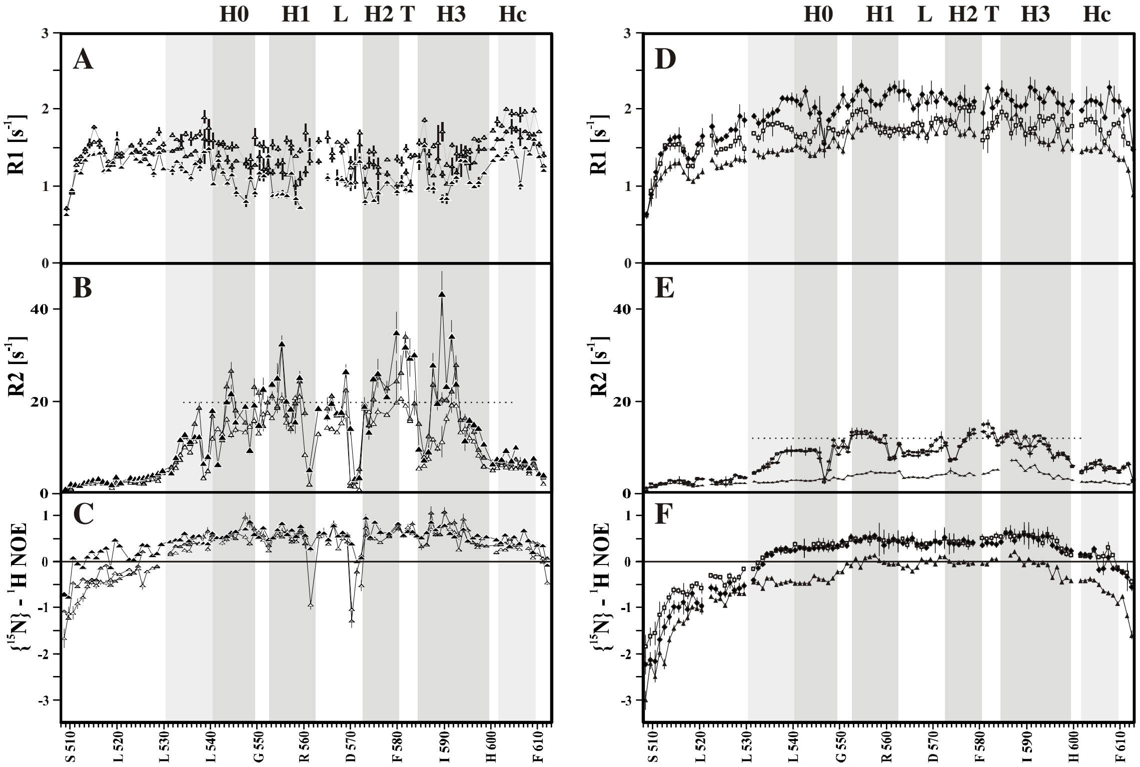

Figure S5. Reduced spectral densities, J(0) (A), J(ω_N) (B), and J(ω_H) (C) determined for the amide backbone nitrogen atoms. Values, represented as a function of rECσ^{70}_4 sequence, were estimated for a 30% (v/v) TFE solution of rECσ^{70}_4 in experiments carried out at 11.7 T (white triangles), 14.1 T (grey triangles) and 18.7 T (black triangles) (25°C, pH 4.6). Grey vertical strips mark helical regions found in the structure of σ^{70}_4 homologues from thermophilic bacteria, and light gray strips mark additional helical regions found in E. coli σ^{70}_4 in the RNAP complex. Experimental errors are denoted by vertical lines. For comparison the analogous data (D-F) obtained previously for low-pH solution at 11.7 T (black triangles), and 10% TFE at 9.4 T (white squares) and 11.7 T (black diamonds) are presented.

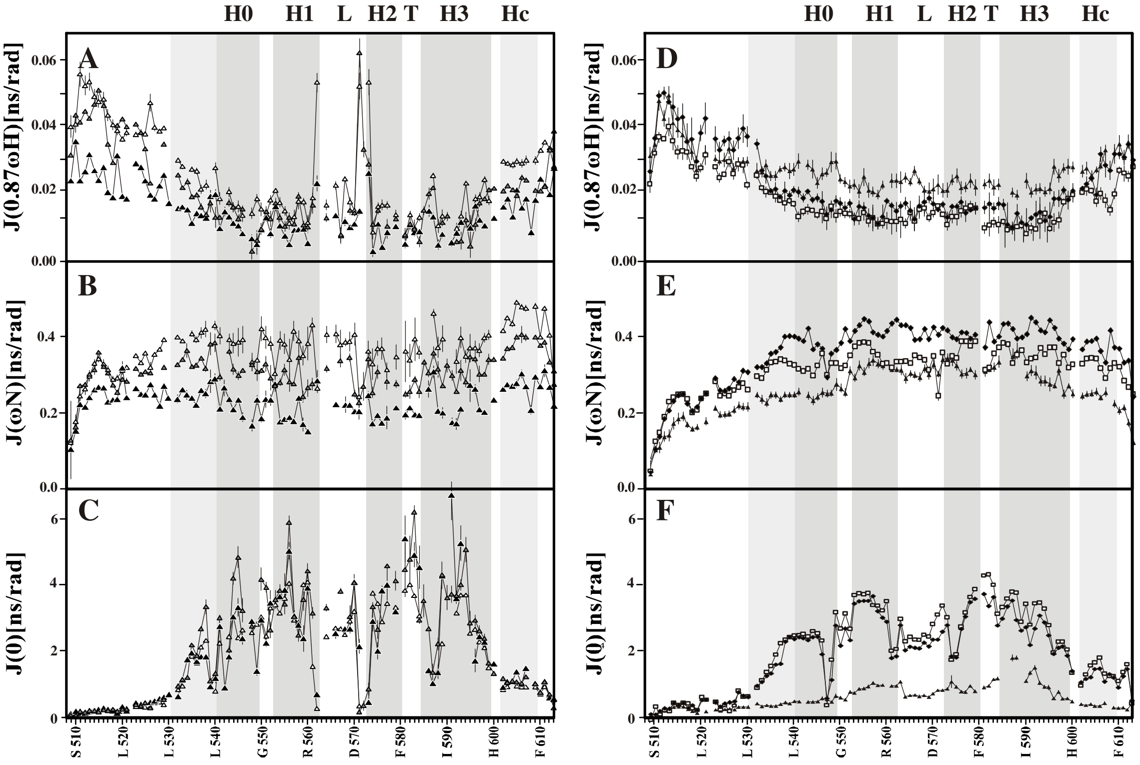

Figure S6. Identified in ^{15}N- and ^{13}C-edited 3D NOESY spectra long-range constraints mapped on the modeled by homology structure of ECσ^{70}_4. The structure of domain 4 of sigma 70 subunit from *Escherichia coli* (red-yellow; ribbon representation) modeled by homology with marked long-range constraints (green tubes)**,** deduced from various NOESY experiments. **The s**ide chains of respective amino acid residues engaged in **a** long-range interaction are marked in blue. The experimental pattern of structural interactions is consistent with the model**e**d spatial arrangement of helical regions.

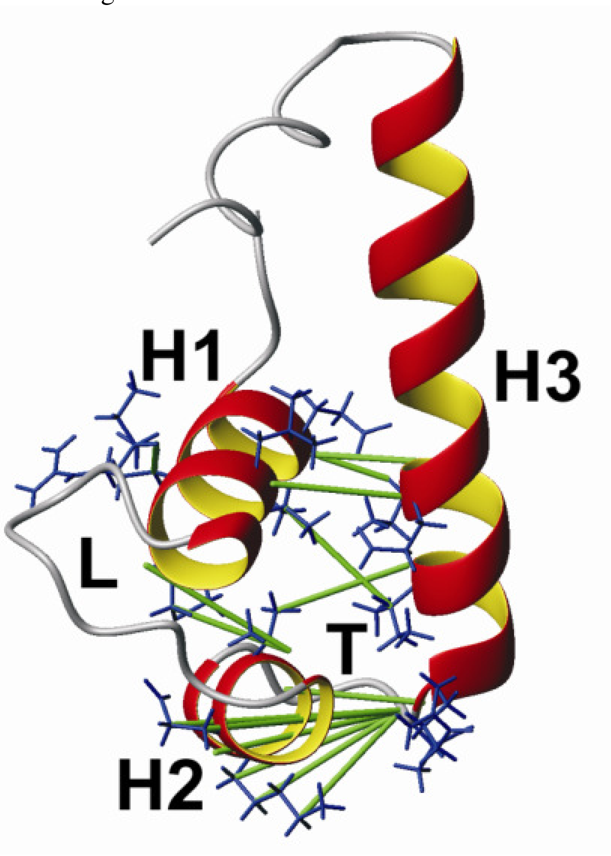

Supplement: Supplementary file 1 — Supplementary material 1 (PDF 537 kb) [file 249_2014_987_MOESM1_ESM.pdf]
